# A Micro Peristaltic Pump-Driven Step-Flow Autoanalyzer: Application to the Determination of Nitrite, Phosphate, and Silicate

**DOI:** 10.1021/acsomega.5c03305

**Published:** 2025-06-20

**Authors:** Su-Cheng Pai

**Affiliations:** 33561 Institute of Oceanography National Taiwan University, Taipei 106319, Taiwan

## Abstract

A simple, fast, and highly convenient autoanalyzer is proposed and demonstrated for the colorimetric measurement of nitrite, phosphate, and silicate in natural waters. The system employs individual micro peristaltic pumps controlled by integrated electric relay modules, allowing the main flow to be regulated in a stepwise manner. Activation is initiated by a single “start” button, which triggers a series of preprogramed actions, including sample loading, reagent mixing, refluxing of residual liquid, pausing for complete color development, and flushing the system with distilled water to reset the baseline to zero. Each measurement cycle is completed within 100 s with mild online heating. Real-time signals are captured via dynamic data exchange (DDE) and transferred to an Excel worksheet for instant calculation and display. Three systems are constructed and demonstrated for the determination of nitrite, phosphate, and silicate. Results indicate that over 99% completeness of the chemical reaction has been achieved with a precision better than ± 0.5% R.S.D. As each system is dedicated to a specific parameter, simplifying maintenance and minimizing troubleshooting, the system is highly convenient for use aboard research vessels or in busy laboratories, catering to users who need immediate measurements in either the “one-touch-one-sample” or “automatic-repeating” mode on a self-service basis.

## Graphical abstract

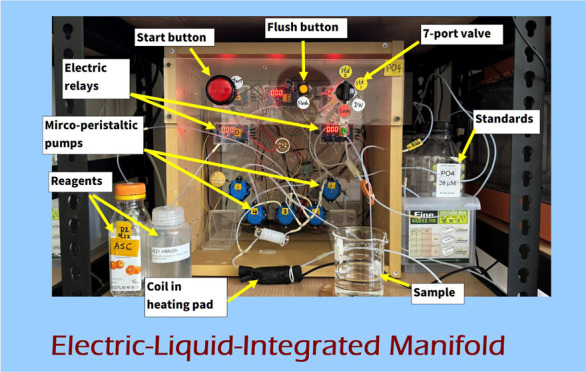

## Introduction

Numerous types of commercial automated instruments are available on the market for various colorimetric determinations. Among them, the air-segmented flow autoanalyzer (SFA),^1–4^ flow injection analyzer (FIA)^5–13^ and sequential injection analyzer (SIA)^14–26^ are probably the most widely adopted. These three techniques and their derivatives were developed at different times and may be roughly regarded as three generations in automated analysis.^27–29^ The basic principle of SFA^1,2^ involves injecting a sample section into a continuous bubble-segmented carrier flow driven by a multichannel peristaltic pump. The main flow merges with the reagent flow(s) and passes through mixing coils on the manifold, where the chemical reaction occurs while traveling toward the detection unit. When the debubbled mixture passes through a narrow-bore flow cuvette in a colorimeter, continuous absorbance is recorded. The peak height is used to calculate the concentration of the analyte. The basic principle of FIA^5^ is quite similar to that of SFA, but it uses narrower tubings and faster flow rates to reduce longitudinal diffusion along the tubular channel. It takes advantage of faster operation and does not require air-segmentation in the carrier flow or a debubbler before the flow reaches the detector. However, the FIA does not measure signals in a steady state. As for the SIA technique,^17,19,20^ the liquids are driven by a syringe pump with a back-and-forth piston in a cylinder, allowing the analysis to be performed sequentially or a step-by-step manner. The consumption of reagents is minimal, since it is not continuously fed. In general, each of the above techniques has its own characteristics, but all have achieved the same analytical goals: reducing manual operation, increasing precision, and facilitating analysts’ ability to handle a large number of samples.

However, in our experience of routinely measuring nutrients (e.g., nitrite, phosphate, and silicate) in natural waters using these three types of instruments, we have encountered some inconveniences with each of the techniques on various occasions, particularly when measurements need to be carried out on board a research vessel. These can be described as inevitable “analytical dilemmas” for automated analysis:

(i) In the pursuit of faster analysis throughput, the completeness of the chemical reaction may be reduced, resulting in lower precision and sensitivity.
(ii) In the pursuit of smaller sample volumes, the sample may subject to dilution by the carrier flow, still resulting in lower precision and sensitivity.
(iii) Increased automation often leads to more complex instrumentation, requiring additional setup, calibration, and maintenance before, during, and after use.
(iv) A sophisticated automated system usually requires a skilled operator, making it less user-friendly for nonexperts.

To address these challenges, we have developed a simple, homemade automated system using cost-effective components (e.g., single-channel micro peristaltic pumps, integrated relay modules, and DC/AC converters, all sourced from commercial Web sites) to assemble a manifold that operates without a carrier flow or a sample injector. The pumps do not run continuously but are individually controlled by electric relay modules, allowing the sample segment to remain in the detector for a complete chemical reaction. Such design may be compatible with the recently advertised “programmable flow injection” technique (pSI),^28–30^ which combines flow and batch modes to achieve a steady-state measurement thus eliminating completely the Schlieren effect. The advantage of the proposed system lies in its low cost and functionality, similar to those of a vending machine. It is always on standby, enabling rapid sample loading, detection, and flushing in a stepwise manner. It is designed for nonexpert users, providing almost instant results.

The selection of nitrite, phosphate, and silicate for demonstration in this study is based not only on our routine requirements but also on their similar analytical procedures (a sample treated with two reagents) and the rapid color formation reactions, which can usually be completed in a very short time at room temperature.

## Instrumentation

### Micro Peristaltic Pump

Five Kamoer micro peristaltic pumps (NKP-DA06 DC24 V) were purchased from the commercial Web site (priced at around U$5 each). Those pumps were powered by adjustable AC/DC converters providing a DC output range between 5 and 24 V. The pumping rate was controlled by the size of the pumping tube as well as the DC voltage. Three sizes of pumping tubes were used: with internal diameters of 0.8, 2, and 3 mm, respectively, which produced a wide flow rate range from 2 to a maximum of 90 mL min^–1^(Figure 1.). The selections of pumping tube material were as follows: silicone rubber for the sampling and flush pumps, whereas biopharmaceutical material (BPT tube, PharMed Saint-Gobain, France) was used for reagent pumps. The ratios between the sample flow and the two reagent flows were designated as 25:1:1. A similar ratio was achieved by choosing pump settings of ID2 mm/24 V and ID0.8 mm/6 V, which produced flow rates of approximately 48 and 2.1 mL min^–1^, respectively. The pump for flushing purpose was set at ID3 mm/24 V, which produced a maximum flushing rate of 90 mL min^–1^.

**Figure 1:**
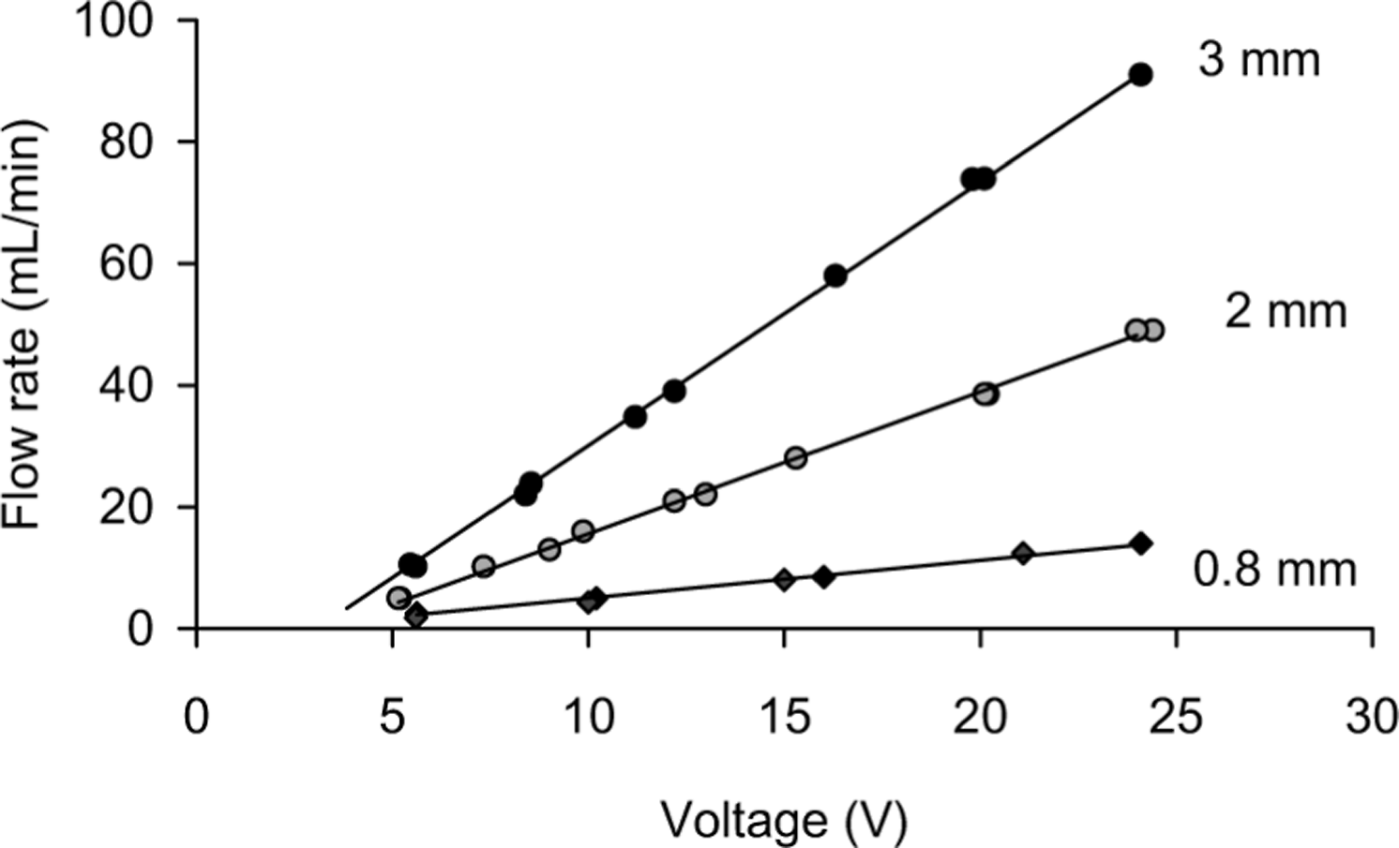
Pumping flow rates of Kamoer NKP-DA06 micro peristaltic pump as decided by tubing size (ID mm) and DC voltage. Three sizes of pumping tubes were tested, from top, ID3 mm, ID2 mm, and ID0.8 mm. This diagram indicates that this pump can provide a wide range of flow rates by choosing appropriate tube size and voltage.

### Integrated Electric Relay Board

Five single-circuit relay modules (DC 5–24 V) were purchased from commercial Web sites (priced at around U$3 each). The module can be configured for various functions. For example, in mode I, a trigger signal switched on the device (e.g., pump or other relays) and then the power was cut off after a designated time period; in mode II, the operation period was delayed for a preset time; and in mode III, the operation was set in a looping mode (*n* = 1 to ∞).

### Teflon Tubings/Coils

The main manifold was constructed using 1–1.5 mm ID Teflon tubes, with wider-bore tubes at the ending part of the tubular channel to accommodate the very high flow rates of up to 90 mL min^–1^. Online mixing coils (30 ∼ 60 cm) wound on 1 cm diameter plastic rod were placed after each addition of the reagent. One of the coils was wrapped in a fabric dry heating pad (10 cm × 20 cm, DC 5 V/5W), which provided a moderate temperature of around 40 °C.

### Rheodyne 7-Port Loading-Selection Valve

An optional device was used to quickly select sample, distilled water, and standards.

### Detector

A Metertech MT-200 spectrophotometer (Metertech, Taiwan) was used. The wavelengths were set at 543, 880, and 400 nm for nitrite, phosphate, and silicate, respectively. A 1 cm path dome-type flow cuvette (Hellma, Germany) with a wide window of 0.45 cm × 1 cm and an inner volume of 450 μL was implemented in the spectrophotometer. The inner chamber had a dome-shaped ceiling so any bubble coming from the inlet flow would be quickly removed and the cuvette would not go dry when the flow stopped.

### The Manifold

It was implemented by assembling the above-mentioned compartments together, as shown in Figure 2. All relays A–E and pumps P1, P4, and P5 were powered by a DC 24 V source, whereas pumps P2 and P3 were supplied by DC 6 V (Figure 3). The detailed settings are given in Table 1. An extra “manual flush button” was implemented for quick flushing the entire tubular channel whenever necessary.

**Figure 2:**
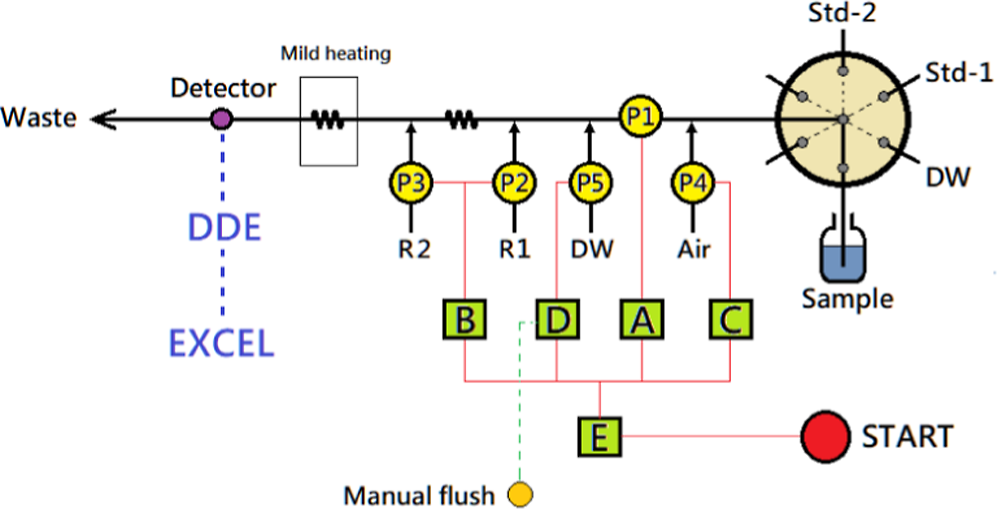
Layout of the manifold for the proposed system. It comprises a 7-port selection valve, 5 individual micro peristaltic pumps (P1–P5) controlled by 5 electric relay modules (A–D), a wide-bore flow cuvette installed in a spectrophotometer, and an online heating device. Absorbance signals are retrieved by DDE and recorded by Excel worksheet. Relays A–D are activated by relay E which can be set for the single or loop mode. Relay E is triggered by a “START” button. Pumps are operated in either the instant or delay mode. A manual flush button is connected to relay D for flushing the system whenever needed.

**Figure 3:**
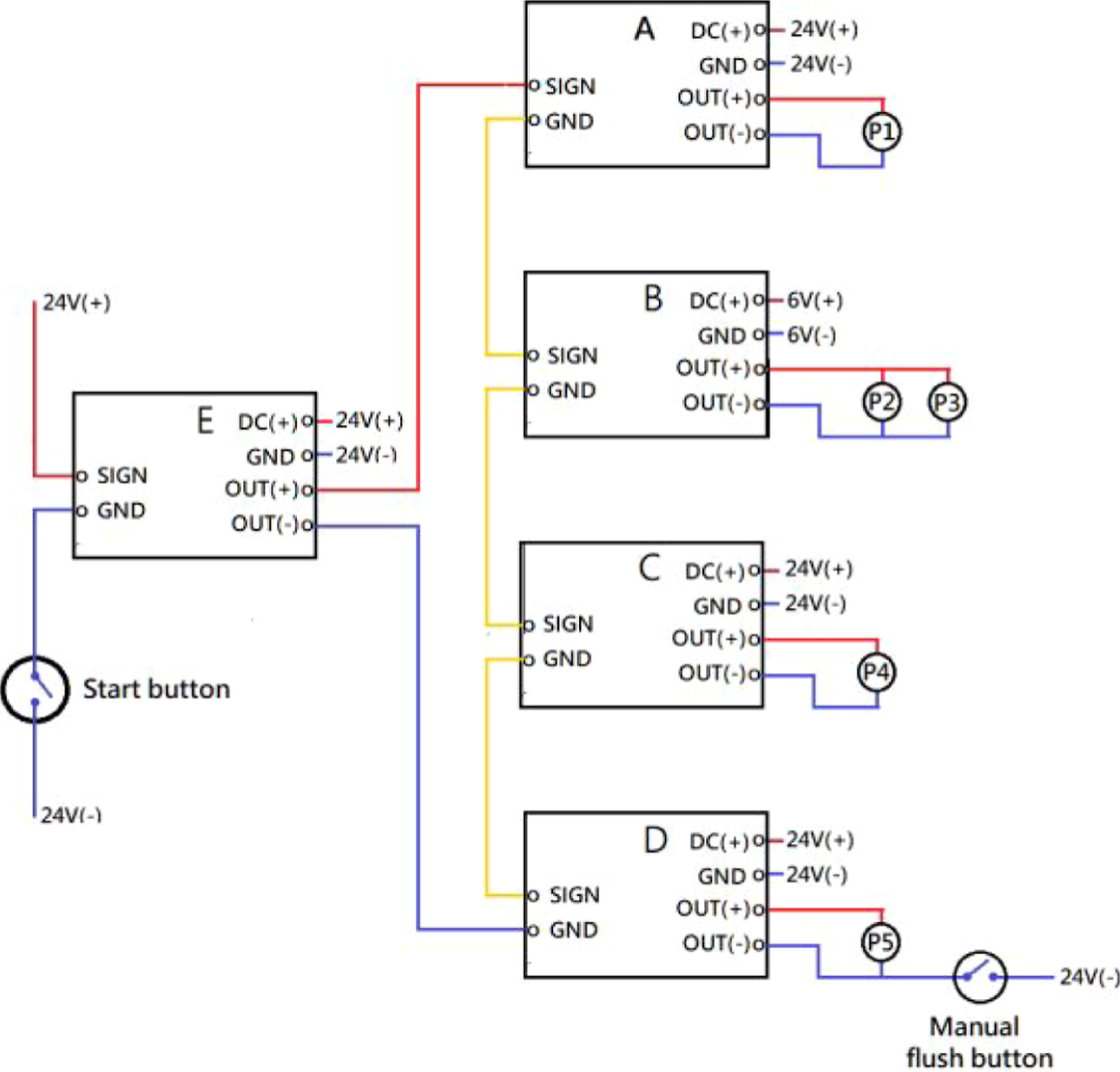
Circuit diagram featuring electric relay modules and micro peristaltic pumps, powered by DC 6 and 24 V supplies. The start button triggers relay E, which in turn activates relays (A–D) to control the activation timing of peristaltic pumps P1, P2, P3, P4, and P5. A manual flush button is connected to P5.

**Table 1:** Setting of Relays and Pumps for a 100 s Measuring Cycle

relay	function	voltage (DC V)	pump	tube ID (mm)	flow rate (mL/min)	mode/operation period (s)	
E	trigger A,B,C,D	24				loop^a^	1 s
A	load sample	24	P1	2	48	instant	0–10 s
B	add reagent R1	6	P2	0.8	2.1	instant	0–10 s
B	add reagent R2	6	P3	0.8	2.1	instant	0–10 s
C	air injection/reflux	24	P4	2	48	delay	9–11 s
D	DW flush	24	P5	3	90	delay	90–100 s

a The loop mode allows single or repetitive measurements.

### Operation Procedure

When a user pushed the “START” button to trigger relay E, it further triggered the other four relays (A–D) to control pumps running either in the instant or delay mode (see Figure 3 and Table 1). The operation for a measuring cycle can be described in five steps (Figure 4). Step 1: Relays A and B were triggered simultaneously, the former controlled the sample loading by pump P1 (operated for 0–10 s), and the latter controlled reagent additions through pumps P2 and P3 (also operated for 0–10 s). Step 2: Just before the loading was finished, pump P4 was switched on by relay C (operated between 9 and 11 s). During the first second (9–10 s), a small section of air would go into the manifold, which evacuated the sample liquid in the pumping tube of P1. Step 3: In the later second (10–11 s), P1/P2/P3 stopped and air refluxed the residual sample solution back to its original bottle. Step 4: After 11 s, all pumps were stopped, and the sample section staying in the cuvette was allowed to develop its color under a static condition for detection. The waiting time was decided depending on the reaction kinetics (for example, the chemical reaction could be nearly completed at 90 s for nitrite, phosphate, and silicate). Step 5: After recording the maximum absorbance, P5 was activated by relay D (operated between 90 and 100 and 10 s) to flush the whole tubular channel with distilled water to bring down the baseline to zero.

**Figure 4:**
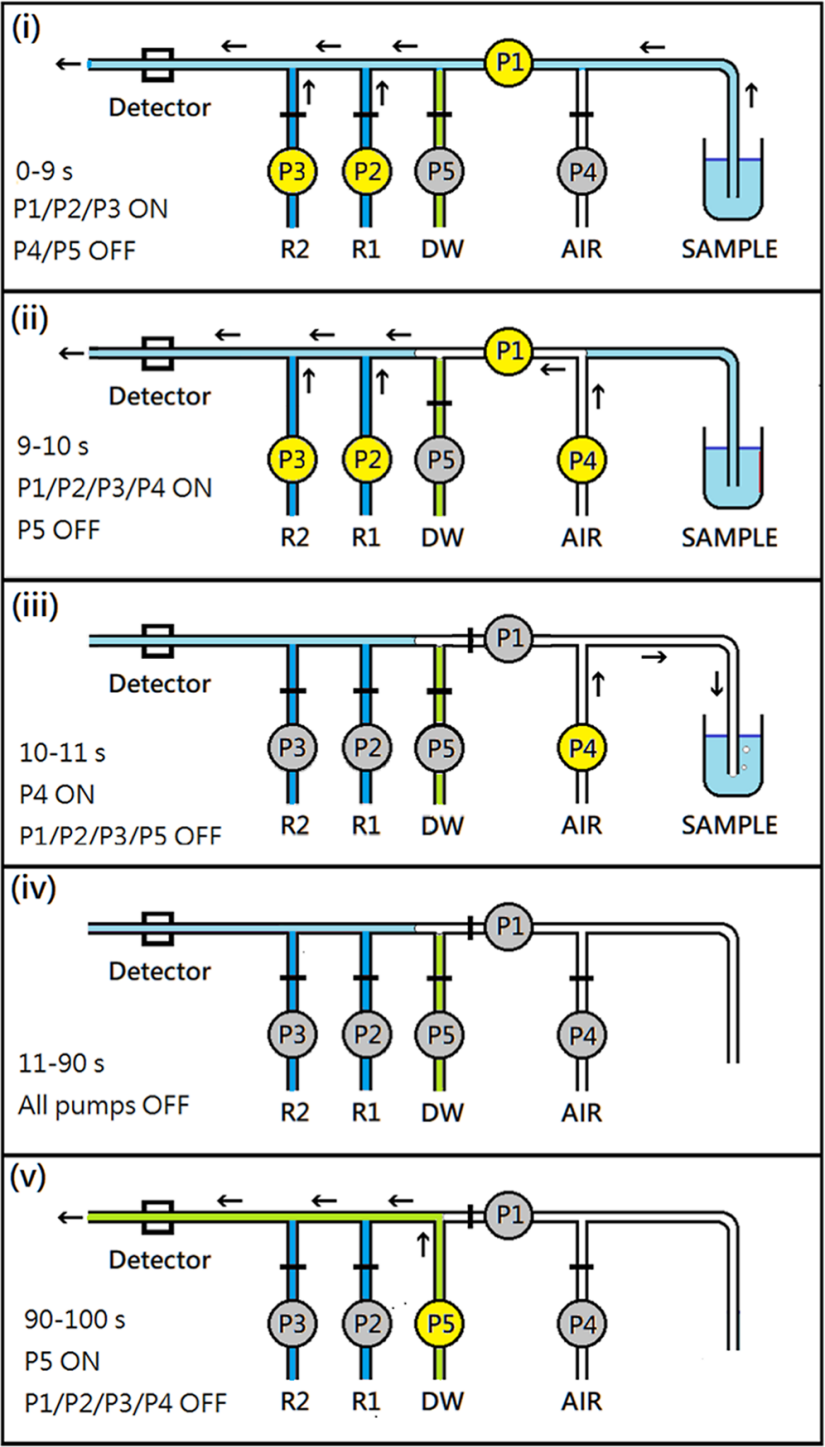
Schematic diagrams illustrating the operating sequence of the manifold during a 100 s program after the user presses the “START” button. The colors represent the following: (blue) sample, (deep blue) reagents, (green) distilled water, (blank) air, (yellow) pump activated, (gray) pump idled, (black arrow) flow direction, and (black bar) indicating flow stoppage. The time control for the five steps is as follows: (i) 0–9 s: P1/P2/P3 ON, (ii) 9–10 s: P1/P2/P3/P4 ON, (iii) 10–11 s: P4 ON, (iv) 11–90 s: all pumps stopped, and (v) 90–100 s: P5 ON. The sample and reagent mixture is retained in the flow cuvette of the detector between 10 and 90 s.

### Signal Retrieving and Filtering by Excel

The real-time absorbance signals from the spectrophotometer were retrieved via dynamic data exchange (DDE) at a baud rate of 19,200 and recorded in an Excel worksheet through an RS-232 port at a rate of one reading per second. We chose “Excel 2003 Edition” for this purpose (note: other later editions have number limits on graphic labels). The real-time signal was continuously written to a fixed Excel cell (e.g., cell position “$C$2”). Simultaneously, a Visual Basic program was executed to copy the incoming value every second, first to a designated cell address (e.g., “B10”) and then moved to the next cell below (e.g., “B11”, “B12”, and so on). In this manner, the Excel worksheet could store accumulated signals over an 8 h period (i.e., 28,800 data points). With this data set, further calculations could be performed instantly, and the real-time recorder trace for a selected time period (e.g., 1 h scale) could be plotted and displayed on the computer screen (see Appendix 1 for details).

### Air Spikes and Software Filtering

When air sections or bubbles passed through the detector, signal spikes occurred. These spikes typically lasted only 1–2 s at high flow rates exceeding 0.8 mL s^–1^ (Figure 5). The spike was identified by the sudden change of absorbance, i.e., ΔAbs/Δ*t* ≥ *x*, where x is the threshold value. These unwanted spikes could be eliminated by software filtering.

**Figure 5:**
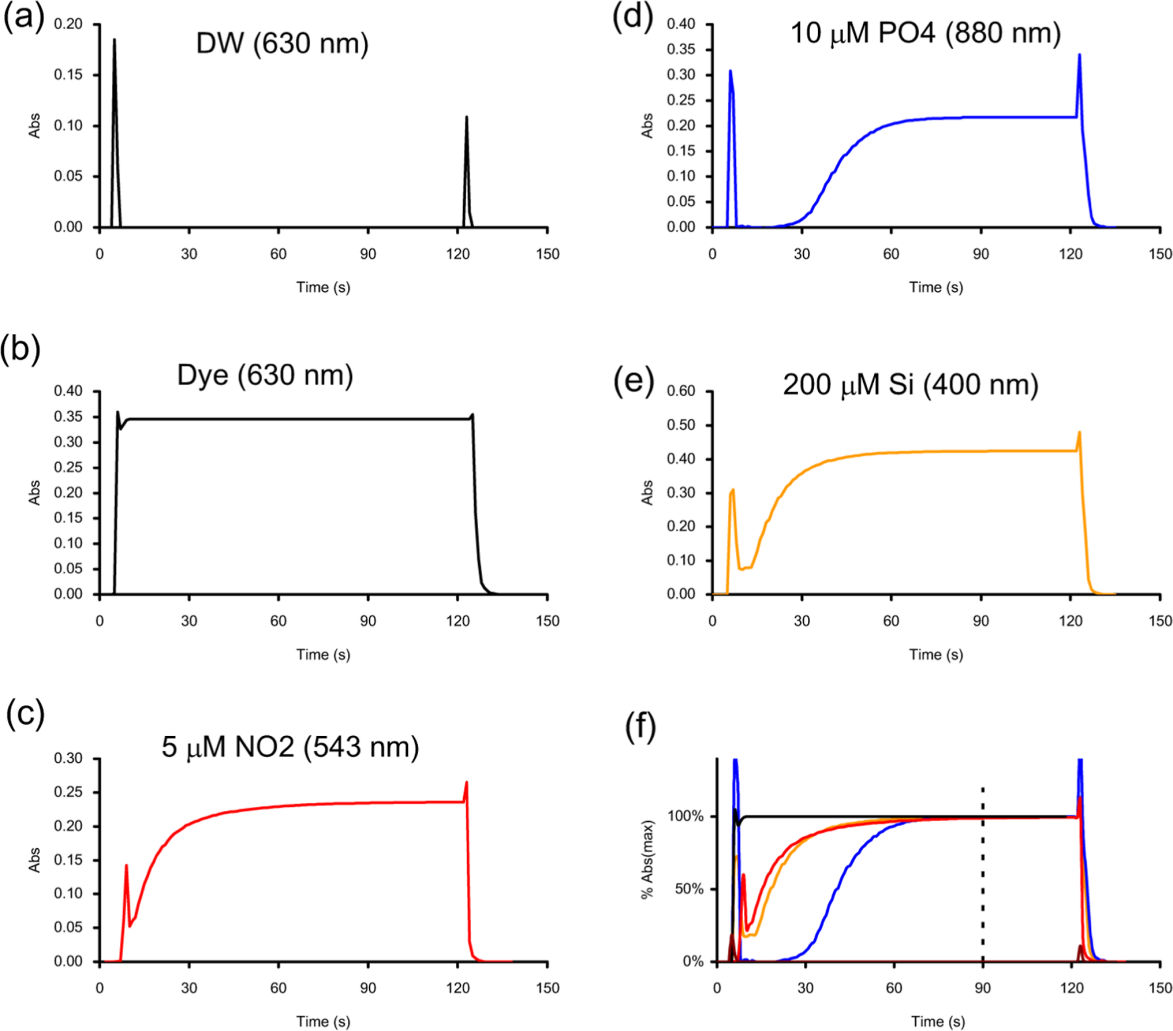
Bubble spikes and peak shapes for (a) distilled water at 630 nm, (b) a dye solution at 630 nm, (c) a 5 μM nitrite solution at 543 nm, (d) a 10 μM phosphate solution at 880 nm, (e) a 200 μM silicate solution at 400 nm, and (f) overlapped curves on a % Abs (max) axis, indicating that 99% reaction completeness is achieved at 90 s. The loading and flushing times were set for 0–10 s and 120–130 s, respectively.

A filtering formula was preprogramed in the Excel cells. For example, if the signal fluctuation exceeded a threshold (e.g., ± 0.050 AU/s), it was regarded as a spike. The neighboring signals within the range of −5 to +5 s were then skipped, i.e., Abs­(*t*) = Abs­(*t*–1), otherwise Abs­(*t*) = Abs­(*t*) (see Appendix 2 for details).

### Baseline Identification

After each measurement, the baseline was checked once the cuvette had been flushed with distilled water, and the flow was stopped. The Excel worksheet utilized the “IF” function to identify the baseline. Three conditions should be met: (i) the average readings of the last 3 s and the readings from 3 to 6 s prior should be equal, (ii) the absorbance should be no greater than 0.000, and (iii) no other baseline mark should appear in the past 50 s. Matching the three conditions, a baseline value was marked on the screen. In the case that the lamp of the spectrophotometer does not warm up enough, causing the baseline to drift, the instrument’s zero was simply reset.

### Peak Labeling

When all flows ceased, the chemical reaction continued to progress and gradually reached a steady state or completion. A previously programed formula was used to determine which reading should be taken. The Excel worksheet utilized the “IF” function to identify a peak summit, based on three conditions: (i) the absorbance variation of the last 10 s should be less than 0.001 or the coherence of the mean absorbance readings from the last 9–16 s and the last 8 s should reach a coherence threshold value (e.g., 0.995), (ii) the signal should be greater than or equal to the reagent blank, and (iii) no other peak should have been marked in the past 70 s. If these three conditions were met, the absorbance reading was deemed a peak summit, and a label with the absorbance value appeared on the chart.

### Concentration Calculation

Once the reagent blank was obtained, it typically remained valid for the entire day. Given that each system was dedicated solely to one parameter, the slope of the calibration curve remained highly stable, with only very small variation (e.g., usually no more than 1% on the same day), and could be regarded as an empirical constant for that system.

The slope value was input manually on the Excel worksheet. The concentration was determined using the formula: Conc. = (Abs - reagent blank)/slope. The user could choose to label concentration or absorbance values onto the peaks on the Excel screen.

## Experimental Section

### Determination of Nitrite

The pink azo dye method^33,34^ was adopted, in the manual protocol, where nitrite reacted with sulfanilamide under acidic conditions to form a diazonium ion which later reacted with the NED reagent to form a final pink azo dye, which was measured at 543 nm. The time for developing a full color could be long if the two reagents were mixed too soon.^34^ To minimize this problem, an extra mixing coil was added to the manifold at the position just after the addition of R1 to make sure the first addition action is well-mixed with the sample. Mild online heating was found very helpful to shorten the time for the complete color formation reaction. Two reagents were prepared as follows:

R1­[HSUL]: 10 g of sulfanilamide was dissolved in 500 mL of 15% HCl.

R2­[NED]: 1.5 g of *N*-1-naphthylethylenediamine was dissolved in 500 mL of DW.

### Determination of Phosphate

The molybdenum blue method^35,36^ was adopted. Phosphate reacted with acidic molybdate to form a yellowish phosphomolybdic acid which was reduced to a dense blue color by ascorbic acid in the presence of antimonyl ion as the catalyst. The color was measured at 880 nm. One of the major concerns was the expiration of the ascorbic acid reagent. It was well understood that ascorbic acid deteriorated quickly in aqueous solution; thus, a freshly prepared solution usually lasted only for a few days. Although a recent report^37^ claimed that the ascorbic reagent remained useable for 50 days if it was stored in the dark in a sealed bag, we have found if the solution was mixed with an antioxidant - sodium sulfite at a weight ratio of 10:1, it could last for up to several months without turning brownish. Sulfite does not interfere with the formation of the phosphomolybdenum blue color. The reagents for the phosphate measurement were prepared as follows:

R1­[HMoSb]: it was prepared by mixing three solutions: (a) 115 mL of conc. sulfuric acid in 250 mL, (b) 10 g of ammonium heptamolybdate tetrahydrate in 200 mL, and (c) 1 g of potassium antimonyl tartarate in 50 mL.

R2­[ASC]: 20 g of l­(+)-ascorbic acid and 2 g of sodium sulfite were dissolved in 500 mL of DW.

### Determination of Silicate

The molybdenum yellow method^38^ was adopted with modifications on the molybdate reagent, which was prepared in ammonia–water for longer stability. Under acidic conditions, silicate reacted with molybdate to form a yellowish compound which could be measured directly at 400 nm. The color formation reaction was very quick, and a steady state could be attained within 70 s. Since phosphate also reacted with molybdenum and produced a similar color at 400 nm, the measured results should be termed [Si + P]. Although adding oxalate could effectively eliminate the phosphate interference,^38,39^ it was not intended to do so as the extra reaction would prolong the analysis time. Instead, we suggested that the [Si] concentration could be obtained by direct subtraction of phosphate interference from the [Si + P] result. Two reagents were prepared as follows:

R1­[H2SO4]: 35 mL of conc. sulfuric acid was poured gradually in DW to make a final volume of 500 mL.

R2­[MoNH3]: 65 g of ammonium heptamolybdate tetrahydrate was dissolved in ca. 400 mL of DW containing 25 mL of 25% w/v ammonia, and the mixture was made up to 500 mL with DW.

## Results and Discussion

### Bubble Spikes and Peak Shapes

A distilled water sample was used to identify bubble signals using the proposed manifold with 0–10 s autoloading at a rate of 48 mL min^–1^ and 120–130 s autoflushing at a rate of 90 mL min^–1^. The absorbance was measured at 630 nm (Figure 5). After the start button was pressed, the first air section passed through the detector at 5 s, causing a spike signal of approximately 0.18. The spike lasted only 1–2 s, after which the absorbance promptly returned to the baseline. When the flush started at 120 s, a second bubble spike of approximately 0.055 appeared at 123 s on the chart, lasting for only 1 s. Repeated tests showed that spike readings were random but all exceeded 0.05 in absorbance. These spikes consistently appeared 5–6 s after sample loading and 3–4 s after flushing. This sharp shape of the spike was attributed to the fast flow rate and the dome-type flow cuvette, which could not trap air bubbles when the flow was stopped.

A blue dye solution (methylene blue, with an absorption maximum at 630 nm) was used to check the physical condition of the manifold (Figure 5). The solution was quickly loaded, reached the detector in 5 s, and provided a > 99% maximum reading within 10 s. A small spike was barely observed, as it was masked by the absorbance signal of the dye. When pump P5 was turned on, the entire tubular channel was rapidly flushed with distilled water at a high flow rate of 90 mL min^–1^. The total flushing volume used was 15 mL. This caused the absorbance to drop to 0.005 in 3 s and return to baseline zero in 6 s. After flushing, no sample liquid remained in the flow system, completely eliminating the “carry-over” problem or “memory effect”. The peak shape resembled a histogram with a minimal tailing.

### Peak Shapes for Nutrients

Similarly, we tested three standard solutions: 5 μM nitrite, 10 μM phosphate, and 200 μM silicate, by measuring absorbance at wavelengths of 543, 880, and 400 nm, respectively (Figure 5).

For the 5 μM nitrite solution, the first spike appeared at approximately 6 s, and the absorbance increased exponentially as the color formation reaction progressed and reached a final reading of 0.237 after 120 s. The reading at 90 s was 0.236, indicating that more than 99% of the reaction was complete. The flushing spike appeared at 123 s, as expected, and the absorbance dropped to zero after an additional 4 s.

For the 10 μM phosphate solution, the first spike appeared 5 s after loading, which was significantly higher than that for nitrite. This difference could be attributed to the high viscosity of the molybdenum reagent, which may have caused a refractive index signal in addition to the air bubble signal. The reaction for the blue phosphate color was initially slow but accelerated after 30 s, reaching a final steady-state absorbance after 80 s. Flushing at 120 s caused a second air spike to appear at 123 s, and the absorbance took another 6 s to return to baseline. The flushing time required was slightly longer than that for dye or nitrite, likely due to the viscosity of the [HMoSb] reagent, which contains very a high concentration of sulfuric acid.

As for the 200 μM silicate solution, the first spike appeared 5 s after activating the loading pump. The color formation started rapidly in an exponential trend and reached 99% completeness at ca. 70 s, slightly quicker than that for nitrite and phosphate. Upon flushing, a small spike signal was seen at 123 s, and the baseline returned to zero 4 s later.

Based on the above findings and for simplicity, the operational programs for the three nutrients were standardized to be identical: 0–10 s for loading, 9–11 s for air injection and refluxing, 11–90 s for waiting, and 90–100 s for flushing. The total duration of each measurement cycle was 100 s with an effective sample trapping time of 80 s in the flow cuvette.

### Bubble Spike Filtering

For each 100 s measurement cycle (Table 1), there were two bubble spikes: one at 5–6 s and another at 93 s after loading, with flushing occurring at 90 s. The former spike was usually higher than the latter, as the air section at the beginning was slightly larger (also included the inner volume of the sample-drawing tube). Such spikes could be identified by calculating the gradient of absorbance and comparing it to a preset threshold value *x*, where *x* can be set to 0.05. This means that if the change in absorbance per second exceeded ± 0.05, it was regarded as a spike at time *t*. In an Excel worksheet, the following formula can be used:

=IF(ABS(Abs(t)−Abs(t−1))>x,1,0)

If Abs­(*t*) was identified as a spike, the neighboring data from Abs­(*t* – 5) to Abs­(*t* + 5) would be filtered out. This function also accounted for the sudden drop in absorbance readings during the flushing stage. As a result, the peak tailing (usually less than 5 s after the appearance of the peak summit) would be removed and the peak would appear sail-shaped with no tail.

A spike-filtering test was conducted for measuring two phosphate solutions (shown in Figure 6). Each measurement included one loading spike and one flushing spike, though the latter might not always be detected as it was likely masked by the high absorbance. After spike-filtering, all peaks appeared distinct without any tailing. However, it should be noted that the filtered peaks appeared wider or “fatter” by 5 s compared to their original shape.

**Figure 6:**
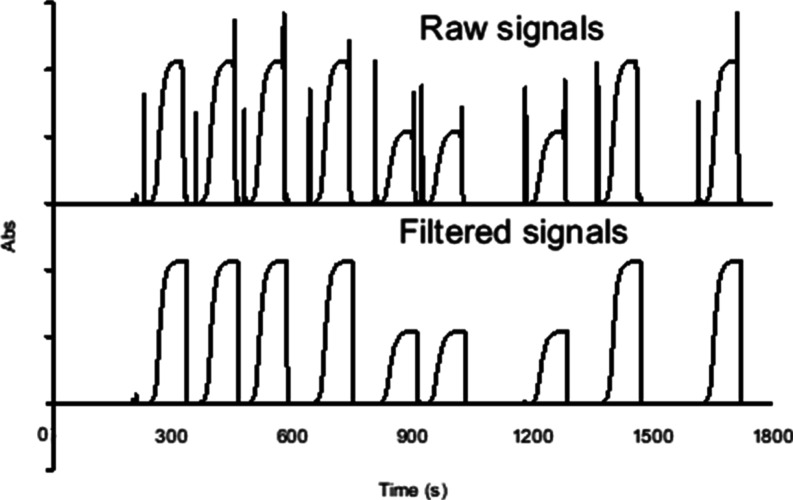
Elimination of bubble spikes from raw signals using software filtering. (Top) raw signals for measuring 10 and 20 μM PO_4_ solutions, showing a loading spike and a flushing spike on either side of the sample peak when air bubbles pass through the cuvette. (Bottom) the spike signals are filtered out, leaving only the nonflowing signals displayed on the computer.

### Overall Performance

A 1 L dye solution was prepared and placed in the loading position, and the reagent bottles were replaced with distilled water. Relay E was set to the “Loop” mode to send a “start” signal to the other relays every 120 s. After pressing the start button, the measurement cycle began and repeated every 2 min, yielding a throughput of 30 peaks per hour (Figure 7.). All peaks were rectangular in shape, each labeled with an absorbance reading taken ∼33 s after loading. The baseline was autochecked after each measurement, with a label of 0.000 displayed after each peak at a position of 109–110 s from loading. The precision was excellent, with the average value for 30 readings being 0.347 ± 0.0005, corresponding to a precision of ± 0.15%. The same pattern was further observed over an additional 4 h period, demonstrating the physical consistency and reliability of the proposed system.

**Figure 7:**
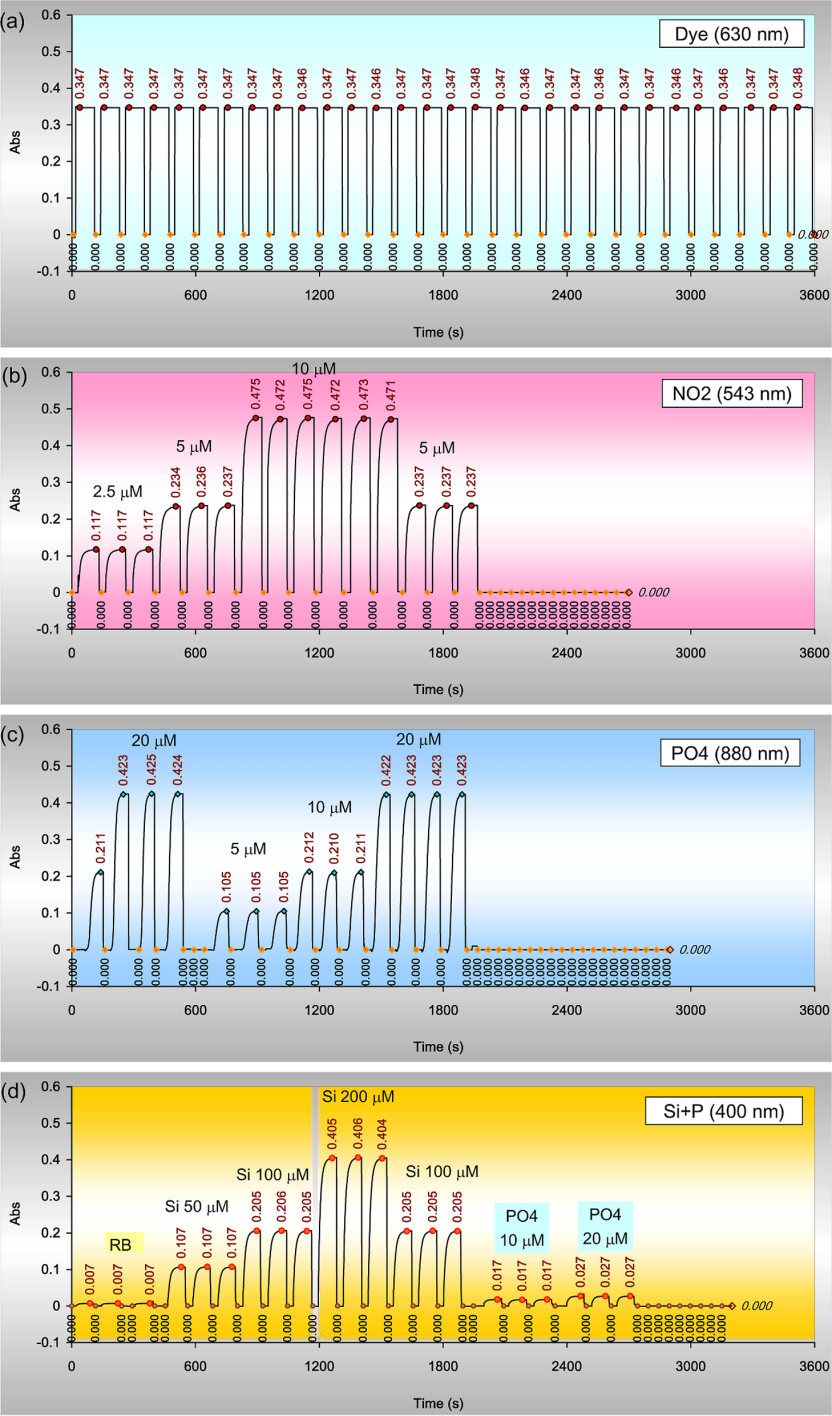
Typical determination tracks of the proposed system. (a) A methylene blue dye solution of arbitrary concentration measured at 630 nm in the autolooping mode every 2 min. (b) Discrete measurements of 2.5, 5, and 10 μM nitrite solutions at 543 nm. (c) Discrete measurements of 5, 10, and 20 μM phosphate solutions at 880 nm. (d) Discrete measurements of the reagent blank, 50, 100, and 200 μM silicate solutions, as well as 10 and 20 μM phosphate solutions at 400 nm. The loading time was 0–10 s, and flush time was 90–100 s.

Three nitrite standard solutions—2.5, 5.0, and 10.0 μM—were tested discretely using the system (Figure 7.). The averaged readings were 0.117 ± 0.000, 0.237 ± 0.0005, and 0.473 ± 0.0016, respectively, with a precision better than ± 0.4% (RSD). The reagent blank was 0.000. All peaks exhibited the expected sail shape with no tailing. The linearity could extend to 20 μM. The calibration slope or absorptivity was 0.0473 μM^–1^, showing no salt effect if the standards were spiked to filtered surface seawater (Table 2.). For samples with very low concentrations (e.g., < 1 μM), a 5 cm light path flow cuvette could be used, but the operation times for loading and flushing would need to change from 10 to 15 s.

**Table 2:** Performance of the Proposed System on the Measurements of Nitrite, Phosphate, and Silicate

parameter	nitrite	phosphate	silicate	phosphate^c^
wavelength (nm)	543	880	400	400
reaction time (s)	90	90	90	90
sensitivity (μM^–1^)				
in DW (1 cm)	0.0473	0.0210	0.00199	0.0010
in DW (5 cm)	0.237	0.105	0.010	0.005
in SW^a^ (1 cm)	0.0473	0.0209	0.00196	0.00095
precision^b^ (R.S.D.)	0.4%	0.5%	0.2%	
linear range (μM)	0–15	0–30	0–500	
throughput (hr^–1^)	30	30	30	

a Standards were spiked to filtered surface seawater.

b Evaluated on 10 μM nitrite/phosphate, 100 μM silicate.

c Interference of phosphate at 400.

Similarly, three phosphate standard solutions were tested using the same system (Figure 7 and Table 2.). The reagent blanks showed no detectable absorbance. The color formation curve followed a logistic pattern, starting slowly but accelerating after 30 s, reaching 99% max at ca. 80 s. The average absorbances for the three standard solutions were 0.105 ± 0.000 for 5 μM, 0.211 ± 0.001 for 10 μM, and 0.423 ± 0.001 for 20 μM. The precision was < ± 0.5%. The reaction rate for the 5 μM solution was slightly slower than that for higher concentrations. Mild online heating was necessary for low concentrations and cold conditions, effectively mitigating this difference. The calibration slope (sensitivity) was 0.0211 μM^–1^, without a significant salt effect. The sensitivity could be increased by 5 folds if a 5 cm long flow cuvette was to be used, but again, the times for loading and flushing would need to be increased.

For silicate measurements at 400 nm, the reagent blank absorbance was 0.007. Three standard solutions (50, 100, and 200 μM) were tested. The color formation curve exhibited an exponential trend and reached a steady state at approximately 70 s. The average absorbance values for these standards were 0.107 ± 0.000, 0.205 ± 0.0004, and 0.405 ± 0.0005 (Figure 7 and Table 2.). After subtraction of the reagent blank, the calibration slope (sensitivity) was 0.00199 μM^–1^. Phosphate also produced a signal at 400 nm. Phosphate solutions of 10 and 20 μM yielded an absorbance of 0.017 and 0.027, after being deducted from reagent blank, giving a slope of 0.0010 μM^–1^—approximately half that of silicate. This indicated that phosphate interference at 400 nm was equivalent to 50% of the silicate signal at the same concentration. For real applications, especially when [Si]≫[P], the raw silicate concentration should be expressed as [Si + P], and the true silicate concentration can be calculated as

[Si](μM)=[Si+P]−0.5×[P]

where [P] is the phosphate concentration, measured separately using an independent system. When standards were prepared in filtered surface seawater, there was a slight decrease (−1.5%) in sensitivity.

It is worth mentioning that according to a recent paper^39^, phosphate interference can be eliminated by adding a mixture of ascorbic acid and oxalic acid. This has not been tested in the current work, but readers can refer to that paper for more details.

### Mild Online Heating

The use of an online dry heating pad (DC5 V, 5 W) in this study may be debatable. In fact, even not using heating, at a room temperature of 25 °C, the reaction completeness for nitrite, phosphate, and silicate could still achieve 96%, 98%, and >99%, respectively, at 90 s. Even though the heating pad, set at 40 °C, could only provide a small amount of heat from the tube wall to the flowing liquid when passing through the mixing coil, experimental results have proved that it can indeed enhance the reaction completeness, especially for cold samples or in cold weather. For this reason, it is always recommended.

### Reagent Consumption and Stability

The volume ratio between the sample and each reagent in this system was 48:2.1. For each measurement cycle, the sampling pump was activated for 10 s (9 s for the loading sample and 1 s for air) at a flow rate of 0.8 mL s^–1^, resulting in an actual uptake volume of 7.2 mL. The consumption of each reagent was only 0.035 mL s^–1^ × 10 s = 0.35 mL. Reagents were typically prepared in 500 mL portions, with each replenishment sufficient for approximately 1400 measurements.

Since the system was designed for long time-span operation (e.g., major checking every week), the effectiveness of the reagents became a major concern. Four out of the six reagents (Table 3) used in this work were highly stable, including [HSUL] for nitrite, [HMoSb] for phosphate, and [H2SO4] and [MoNH3] for silicate. The ascorbic acid reagent [ASC] remained effective for more than 3 months at room temperature if it was added with the deoxidizing agent (sodium sulfite). As for the [NED] reagent for nitrite, it gradually changed from a pale purple to a dense purple color during storage but remained effective for 1 month at room temperature if stored in a dark bottle.

**Table 3:** Stability of Reagents at Room Temperature

parameter	label	abbv	stability
nitrite	R1	[HSUL]	>2 years
	R2	[NED]	1 months
phosphate	R1	[HMoSb]	>2 years
	R2	[ASC]	>3 months
silicate	R1	[H2SO4]	very long
	R2	[MoNH3]	>2 years

## Conclusion

The proposed system, named the “Step-Flow Autoanalyzer” (StepFA), does not appear to fit into any existing category of automated methods.^22,27,29^ Instead, it can be described as a hybrid approach that integrates key characteristics and advantages of air-segmented flow autoanalyzers (SFAs), flow injection analyzers (FIAs), sequential injection analyzers (SIAs), and even the programmable flow injection (pSI)^30–32^ while achieving the same analytical goals with much simpler components—without a carrier flow or injector. Despite its simplicity, it effectively addresses the analytical challenges outlined in the introduction.

This novel system does not prioritize small sample sizes, miniature manifolds, or capillary flow cuvettes. Instead, it utilizes a relatively large sample volume (7.2 mL), high flow rates (48–90 mL min^–1^), a large flushing volume (15 mL), and a wide-bore cuvette for detection, emphasizing nearly 100% reaction completeness without encountering the Schlieren effect. Its application to the determination of nitrite, phosphate, and silicate has demonstrated satisfactorily its efficiency and reliability for routine analysis.

A notable merit of this work is the introduction of an air injection-reflux function, which effectively isolates both sides of the sample section within the tubular channel. By combining this with a subsequent quick flushing process, the troublesome memory effect can be completely eliminated. Regarding the bubble issue, since only the “stop-flow” absorbance signals are considered, spikes caused by air bubbles can be readily filtered out using data retrieval software.

The proposed automated system has several other advantages:

1. For a measurement cycle, each individual pump works only for no more than 10 s, thus prolongs the lifetime of pumps and pumping tubes. When not in use the system is idled in the “standby” mode for the whole day and can be activated right away once the START button is pushed.
2. In the traditional system for phosphate measurement, the blue color tends to coat on the cuvette glass window after a long run and thus causes baseline drifting. This problem is solved by quick and large volume flushing after every measurement.
3. Each system is implemented exclusively for one parameter. Once a daily check on reagent blank and calibration is done, the sensitivity does not change much on the same day.
4. For the system maintainer, it is almost trouble-free, his/her daily work is probably just turning on the power, checking the volume of each bottle, and carrying out the calibration. When the day work is finished, just turn off the power. That is, 10 min to activate the whole system and 1 s to shut down. Releasing pumping tubes or any extra cleaning may not be necessary.
5. The homemade manifold is not difficult to assemble and the cost is inexpensive, yet it is highly versatile and reliable, and the users can modify the manifold or operation program readily to suit many other analytical purposes.

## Supplementary Material



## Notes

Method for retrieving real-time signals using an Excel worksheet and method for software filtering in Excel (PDF)
