# Supplementary material for: A Micro Peristaltic Pump-Driven Step-Flow Autoanalyzer: Application to the Determination of Nitrite, Phosphate, and Silicate
Source: ACS Omega. 2025 Jun 20;10(25):27491–500. doi: 10.1021/acsomega.5c03305 (PMC12224094; doi:10.1021/acsomega.5c03305)

A micro-peristaltic-pump-driven step-flow autoanalyzer: Application to the determination of nitrite, phosphate and silicate

Su-Cheng Pai
Institute of Oceanography, National Taiwan University, Taipei, Taiwan

Appendix 1

Record Abs by Excel every second using Microsoft Visual Basic module

1. Create three modules named “start”, “record” and “timer”.
2. Connect spectrophotometer RS-232 and put the real-time data in cell [C2]
3. Execute the module “start”
4. The Excel will write incoming data starting from cells [A10],[B10], then [A11],[B11], …and so on.
5. Press [ESC] to stop

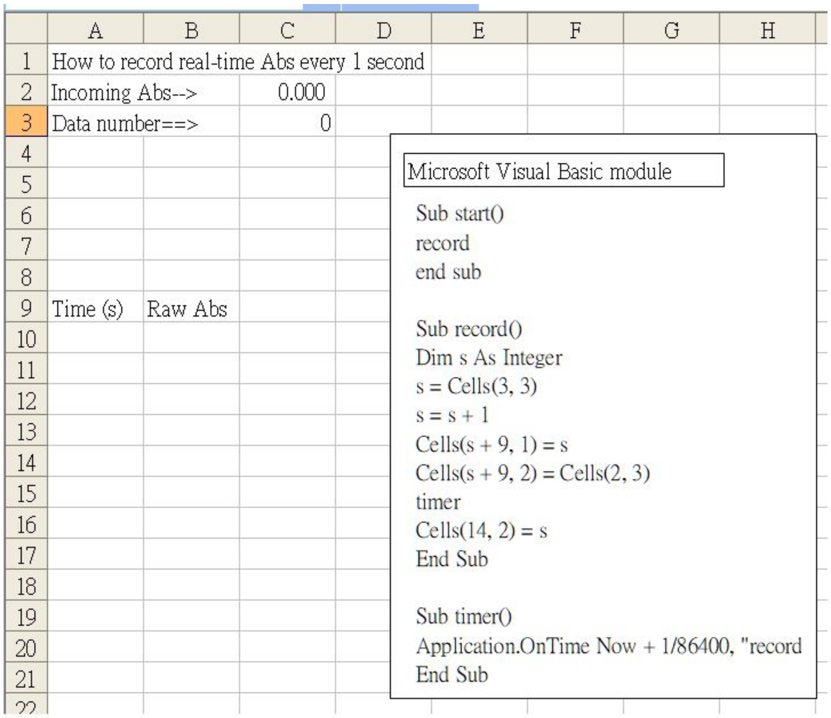

	A	B	C	D	E	F	G	H
1	How to record real-time Abs every 1 second							
2	Incoming Abs-->		0.000					
3	Data number==>		0					
4								
5								
6								
7								
8								
9	Time (s)	Raw Abs						
10								
11								
12								
13								
14								
15								
16								
17								
18								
19								
20								
21								
22								

Microsoft Visual Basic module

```
Sub start()
record
end sub

Sub record()
Dim s As Integer
s = Cells(3, 3)
s = s + 1
Cells(s + 9, 1) = s
Cells(s + 9, 2) = Cells(2, 3)
timer
Cells(14, 2) = s
End Sub

Sub timer()
Application.OnTime Now + 1/86400, "record
End Sub
```

Appendix 2

Software filtering by Excel worksheet to eliminate unwanted spike signals

For example,

1. The Excel has recorded a series of data from [B10] to [B49]
2. Define a threshold value in Cell [C3].
3. In the Cell [C10] input =IF(ABS(B9-B10)>B3,1,0), then copy it down.
4. In the Cell [D10] input =IF(SUM(C5:C14)>0,1,0), then copy it down.
5. In the Cell [E10] input =+IF(D9>0,E8,B9), then copy it down.
6. The original peak shape has two spikes at 10 and 32 s, after "is spike?" and "skip?" judgements, the filtered peak appears between 15 and 37 s, in a square shape.

	A	B	C	D	E
1	Software filtering of spike signals				
2	Incoming Abs=>		0.000		
3	Threshold	0.050	(for identify a spike)		
4	Time(s)	Raw abs	Is spike?	Skip?	Corr abs
5					
6					
7					
8					
9					
10	1	0.000	0	0	0.000
11	2	0.000	0	0	0.000
12	3	0.000	0	0	0.000
13	4	0.000	0	0	0.000
14	5	0.000	0	1	0.000
15	6	0.000	0	1	0.000
16	7	0.000	0	1	0.000
17	8	0.000	0	1	0.000
18	9	0.000	1	1	0.000
19	10	0.114	0	1	0.000
20	11	0.085	0	1	0.000
21	12	0.085	0	1	0.000
22	13	0.085	0	1	0.000
23	14	0.085	0	1	0.000
24	15	0.085	0	0	0.085
25	16	0.085	0	0	0.085
26	17	0.085	0	0	0.085
27	18	0.085	0	0	0.085
28	19	0.085	0	0	0.085
29	20	0.085	0	0	0.085
30	21	0.085	0	0	0.085
31	22	0.085	0	0	0.085
32	23	0.085	0	0	0.085
33	24	0.085	0	0	0.085
34	25	0.085	0	0	0.085
35	26	0.085	0	0	0.085
36	27	0.085	0	1	0.085
37	28	0.085	0	1	0.085
38	29	0.085	0	1	0.085
39	30	0.085	0	1	0.085
40	31	0.085	1	1	0.085
41	32	0.142	1	1	0.085
42	33	0.021	0	1	0.085
43	34	0.005	0	1	0.085
44	35	0.002	0	1	0.085
45	36	0.000	0	1	0.085
46	37	0.000	0	1	0.085
47	38	0.000	0	0	0.000
48	39	0.000	0	0	0.000
49	40	0.000	0	0	0.000
50					

=IF(ABS(B9-B10)>B3,1,0)

=IF(SUM(C5:C14)>0,1,0)

=+IF(D9>0,E8,B9)

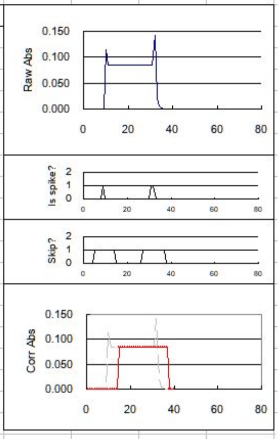

Supplement: Supplementary file 1 [file ao5c03305_si_001.pdf]
